# Facile Labeling of Sieve Element Phloem-Protein Bodies Using the Reciprocal Oligosaccharide Probe OGA*^488^*

**DOI:** 10.3389/fpls.2022.809923

**Published:** 2022-02-10

**Authors:** Pakeeza Azizpor, Lucy Sullivan, Aedric Lim, Andrew Groover

**Affiliations:** US Forest Service, Pacific Southwest Research Station, Davis, CA, United States

**Keywords:** P-protein, phloem, sieve element, phloem feeding insect, phloem fungal

## Abstract

Sieve elements of many angiosperms contain structural phloem proteins (P-proteins) that can interact to create large P-protein bodies. P-protein bodies can occlude sieve plates upon injury but the range of functional and physiological roles of P-proteins remains uncertain, in part because of challenges in labeling and visualization methods. Here, we show that a reciprocal oligosaccharide probe, OGA^488^, can be used in rapid and sensitive labeling of P-protein bodies in Arabidopsis, poplar, snap bean and cucumber in histological sections. OGA^488^ labeling of knockouts of the two Arabidopsis P-protein-encoding genes, *AtSEOR1* and *AtSEOR2*, indicated that labeling is specific to AtSEOR2. That protein bodies were labeled and visible in *Atseor1* knockouts indicates that heterodimerization of AtSEOR1 and AtSEOR2 may not be necessary for P-protein body formation. Double labeling with a previously characterized stain for P-proteins, sulphorhodamine 101, confirmed P-protein labeling and also higher specificity of OGA^488^ for P-proteins. OGA^488^ is thus robust and easily used to label P-proteins in histological sections of multiple angiosperm species.

## Introduction

Phloem sieve elements are responsible for transport of phloem sap containing photoassimilates, nutrients, signaling molecules, and hormones by bulk flow under pressure (Lucas et al., 2013; Knoblauch et al., 2016). This pressurized conduit system is fundamental to plant function and survival, but is susceptible to sap loss upon mechanical injury (Knoblauch and van Bel, 1998). Another danger is posed by phloem-feeding insects and invasive fungal hyphae that access the nutritious phloem sap (Knoblauch and van Bel, 1998; Kehr, 2006; Will and van Bel, 2006). Plants have evolved strategies to circumvent these threats. While incompletely understood, proteinaceous bodies within sieve elements represent one such mechanism that plays roles in safeguarding the phloem.

Phloem-protein (P-protein) bodies have long been recognized as conspicuous ultrastructural features within angiosperm sieve elements (Esau and Cronshaw, 1967). These proteinaceous aggregates can take various forms depending on species and developmental stage (Cronshaw, 1981; Evert, 1990). In general, for intact sieve tubes P-protein bodies do not occlude sieve plate pores and are attached to the plasma membrane to facilitate unobstructed flow within the sieve tubes and across sieve plates (Froelich et al., 2011). Upon injury, P-protein aggregates can be observed at the sieve plates, suggesting that they play a direct role in occluding sieve plates and reducing phloem sap loss (Ernst et al., 2012). Additional potential roles attributed to P-proteins include inhibiting phloem feeding insects (Kehr, 2006; Medina-Ortega and Walker, 2013) and pathogens (Pagliari et al., 2017). However, experimental tests of these various functions proposed for P-proteins have not been conclusive (Knoblauch et al., 2014).

In Arabidopsis, Arabidopsis thaliana Sieve Element Occlusion-Related 1 (*AtSEOR1*) and *AtSEOR2* have previously been shown to encode P-proteins that heterodimerize (Anstead et al., 2012). Live imaging of labeled AtSEOR1 in intact Arabidopsis roots revealed protein structures that varied with developmental stages, including spherical agglomerates in sieve elements shortly after elongation, and filamentous structures later in development (Froelich et al., 2011). This same study combined imaging with matched flow and velocity measurements to conclude that the labeled P-protein bodies did not impede flow in the phloem (Froelich et al., 2011). Mutations in either *AtSEOR1* or *AtSEOR2* resulted in the loss of immunolocalization signals using an antibody recognizing filamentous protein bodies (Anstead et al., 2012). GFP-tagged AtSEOR1 could complement *Atseor1* loss of function mutants but was not able to complement the ability of *Atseor2* loss of function mutants to form filaments. A similar result was observed with GFP-tagged AtSEOR2 failing to complement *Atseor1* mutants. However, while filaments failed to form or label, globular bodies did label within sieve elements for both complementation tests. SEOR-like proteins have also been described in poplar, and are associated with spherical P-proteins that do not respond to phloem wounding (Mullendore et al., 2018).

Efficient methods for visualizing P-proteins have been challenging. The pressurized environment of interconnected sieve elements within sieve tubes results in the effect of injury propagating long distances, and potentially disrupting ultrastructural features found in uninjured phloem. Early observations using transmission electron microscopy were often misleading, as injury and fixation during preparation for microscopy could lead to large changes in the appearance and location of P-protein bodies, which were often observed occluding sieve plates. More advanced methods have provided visualizations that are more faithful to the true appearance *in planta* (Truernit, 2019). However, many of these techniques are technically challenging and may be limited to plant species that can be transformed. More general methods for staining P-proteins have been described, but typically are limited to sectioned material or require some degree of dissection to provide access of dyes to sieve elements. For example, Peters et al. (2006) reported that testing of numerous commercially available dyes failed to identify a compound that uniquely labeled the specialized P-protein bodies (forisomes) of *Vicia faba*. However, sulphorhodamine 101 (SR101) was successfully used to label forisomes in partially dissected *V. faba* leaflets, despite limited membrane permeability of the dye (Peters et al., 2006).

A reciprocal oligosaccharide probe was previously described that shows highly selective binding for chitosan, a product of chitin deacetylation (Mravec et al., 2014). The oligosaccharide probe, OGA^488^, has a structure that aligns opposing charges of carboxyl groups on the probe and chitosan, and carries an Alexa Fluor 488 fluorescent label. The probe was shown to have high binding affinity for chitosan-containing portions of fungal cell walls, as well as chitosan in insect exoskeletons (Mravec et al., 2014). Plants have receptors to detect chitin, which is a well characterized elicitor of plant immune responses (Sánchez-Vallet et al., 2015). However, plants do not produce chitin and it is not a component of plant cell walls (Lee et al., 2011).

In this report, we demonstrate OGA^488^ labels P-protein bodies in the phloem of four Angiosperm species. We present a simple method for labeling of fresh tissues and show that labeling in Arabidopsis is specific to AtSEOR2. We discuss biological interpretations of the binding of the chitin-mimic probe by P-protein bodies in the phloem.

## Materials and Methods

Seeds were obtained from the Arabidopsis Biological Resource Center, United States (ABRC) for *Arabidopsis thaliana* T-DNA insertion lines in the Columbia background for GABI-KAT-609F04 (AtSEOR1 knockout, *Atseor 1-1*, AT3G01680) and SALK 148614C (AtSEOR2 knockout, *Atseor 2-1* AT3G01670). Wild type *A. thaliana* ecotype Columbia was a gift from S. Harmer, University of California Davis. Homozygous knockouts were identified by screening of seedlings from individual selfed plants carrying the T-DNA of interest. Arabidopsis seedlings were grown at 21°C under continuous light on vertical petri plates containing Murashige-Skoog medium (Caisson Labs, MSP09-50LT) with 1% sucrose and 0.8% agar. DNA was extracted from bulks of 10 plants per plate using DNeasy Plant Mini Kit following the manufacturer’s protocol (Qiagen, Germantown MD United States). Homozygosity of plant lines was determined using PCR and line-specific primers. DNA from *Atseor 1-1* plants was amplified using flanking left (5’-CTCGCAACATTTCAGTGAACC-3’) and flanking right (5’-CTAGGGGTAGGTGGAAACTGC-3’) primers (Anstead et al., 2012) that amplify across the T-DNA insertion site to cull lines heterozygous or lacking the T-DNA. An additional amplification with a T-DNA-specific right primer (5’-CAGAACTCGCCGTAAAGACTG-3’) and the flanking left primer was used to detect the presence of the T-DNA insert. The same process was carried out for *Atseor 2-1*, using flanking left (5’-CTGATGATCACCATGTTGCTG-3’) and flanking right (5’-TCTCCGAAACTTCCATAAACG-3’ primers), and a T-DNA-specific left primer (5’-CAACCCTATCTCGGGCTATTC-3’) in combination with the flanking right primer. All PCR amplifications were carried out on a MyCycler Thermal Cycler (Bio-Rad, Hercules, CA) using 92° for 30 s, followed by 40 cycles of 92° for 15 s, 57° for 30 s, 72° for 70 s, and a final elongation at 72° for 5:00 min. PCR products were run on 1.2% (w/v) agarose gels at 70 V for 1.5 h. All amplifications were carried out a minimum of three times with identical results.

For staining and imaging, each experiment used a minimum of three homozygous T-DNA insertion mutants from the *Atseor 1-1, Atseor 2-1*, and wildtype (Columbia) plants that were grown in soil at 21°C under continuous light. Similarly for each experiment, six ramets of clone *Populus tremula* × *P. alba* INRA 717-1B4 (Mader et al., 2016), and six plants each of *Phaseolus vulgaris* and *Cucumis sativus* were grown in soil at 21°C under continuous light, supplemented with fertilizer (Miracle-Gro^®^ All Purpose Plant Food) every 2 weeks following the manufacturer’s instructions. Inflorescence stem segments of bolting *Arabidopsis thaliana* Col-0, *Atseor 1-1*, and *Atseor 2-1* plants were taken by cutting 2–5 cm long sections approximately 1.5 cm above the base of the stem using a razor blade. Stem sections of *P. tremula* × *P. alba* (717) hybrid were harvested from the first fully expanded internodes with secondary growth. Young stems of *P. vulgaris* and *C. sativus* were likewise harvested. Prior to sectioning, Arabidopsis stem samples were embedded in 5% agarose gel. 50 μm thick sections of Arabidopsis, INRA 717-1B4 poplar hybrids, *P. vulgaris* and *C. sativus* were prepared using a vibrating microtome (Leica Vibratome Series 1000) and placed in 50 mM MES buffer pH 5.7.

For staining with reciprocal oligosaccharide probe OGA^488,^ a stock solution of 1 mg/ml OGA^488^ was diluted 1:1,000 in 50 mM MES pH 5.7 as previously described (Mravec et al., 2014). Sections were placed in staining buffer to incubate for 1 h in the dark at room temperature. Samples were then transferred into 1.5 ml 50 mM MES buffer to dilute unbound stain for 0.5–1 h, and then removed using a paint brush to make wet mounts slides with 50 mM MES buffer for microscopy. The used staining solution was stored in the dark at –80°C and could be reused at least 3–4 times without noticeable degradation of signal. For double labeling with SR101 (Sigma-Aldrich S7635), sections were first stained with OGA^488^ for 1 h as above, that solution was removed, and then sections were stained with SR101 at 10 μg/ml in 50 mM MES for 1 h. Sections were then washed twice with 50 mM MES and then imaged. Confocal laser scanning microscopy images were obtained with a Zeiss LSM 710 with 488 nm excitation and a 493–552 nm emission for OGA^488^ and excitation 560 and 611 nm emission for SR101. The same microscope settings were used for all images compared within individual experiments. All experiments were carried out a minimum of three times.

## Results

**FIGURE 1:**
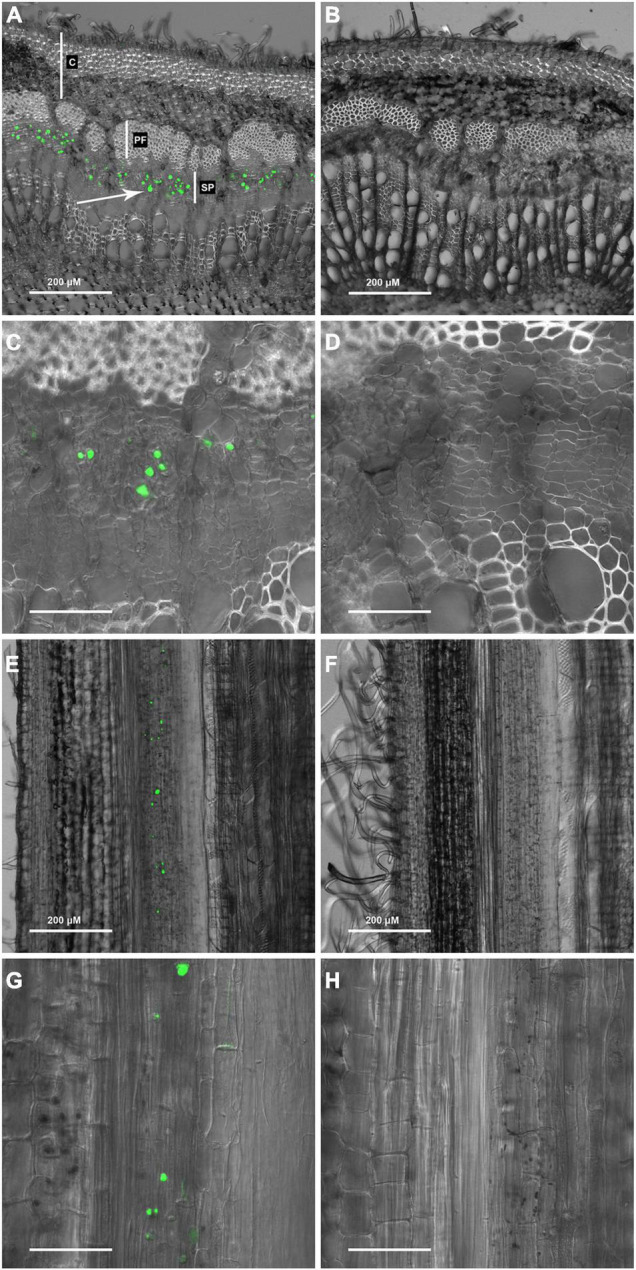
OGA ^488^ labeling of P-proteins (arrows) in *P. tremula* × *P. alba* (717) transverse stem sections. **(A)** 717 stem transverse section labeled with OGA ^488^ C, cortex; PF, phloem fibers; SP, secondary phloem. **(B)** 717 stem transverse section with no probe (control). **(C)** 717 stem transverse section labeled with OGA ^488^. **(D)** 717 stem transverse section with no probe (control). **(E)** 717 stem longitudinal section labeled with OGA ^488^. **(F)** 717 stem longitudinal section with no probe (control). **(G)** 717 stem longitudinal section labeled with OGA ^488^. **(H)** 717 stem longitudinal section with no probe (control). Scale bars: **(C,D,G,H)** = 50 μm.

Toward the goal of establishing new probes for visualizing wood formation in trees, the reciprocal oligosaccharide probe, OGA^488^, was evaluated as a potential negative control for use with other fluorescently labeled oligosaccharide probes recognizing cell wall polysaccharides. Because OGA^488^ is specific for chitosan, which is not a component of plant cell walls, our expectation was a negative result. Fresh cross sections of poplar stems were prepared and probed in 50 mM MES with 1 μg/ml OGA^488^, rinsed and imaged using confocal laser scanning microscopy (see section “Materials and Methods”). Surprisingly, distinct labeling of globular bodies within the secondary phloem was seen in OGA^488^ probed sections (Figure 1A), which were not seen in control sections without OGA^488^ (Figure 1B). Higher magnification images showed that OGA^488^ signal was confined to a subset of individual cells within the secondary phloem with relatively small diameters, consistent with sieve elements (Figure 1C), while control sections showed no signal (Figure 1D). Similarly, longitudinal sections of poplar stems revealed OGA^488^ labeling in elongated cells within the secondary phloem, typically with one or a few rounded bodies labeled within each cell (Figure 1E) with no background staining in sections lacking OGA^488^ label (Figure 1F). Images of longitudinal sections further informs interpretation of cross sections, where the plane of optical sectioning limits detection of OGA^488^ signal above or below the imaging plane. While signals detected in cross sections appear to fill the cell lumen or labeled cells, in fact signal does not extend throughout the length of the cell, and the relative size of the labeled bodies reflect not only their size but also plane of section. Higher magnification images of longitudinal sections probed with OGA^488^ further confirmed the presence of the rounded bodies, but also revealed labeling of more disbursed bodies within the cell, sometimes associated with the basal ends of elongated cells (Figure 1G), while control sections lacked signal (Figure 1H). These results are reminiscent of the morphology of P-proteins previously presented for poplar (Mullendore et al., 2018).

**FIGURE 2:**
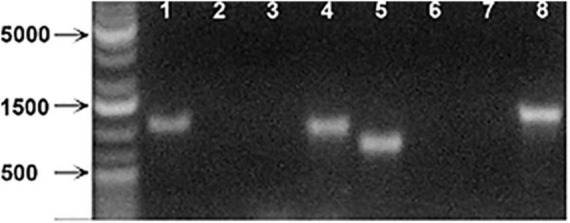
PCR analysis of Arabidopsis SEOR insertion line mutants. (Lane 1) *Atseor 1-1* with *Atseor 1-1* specific T-DNA primer and flanking primer. (Lane 2) WT with *Atseor 1-1* specific T-DNA primer and flanking primer. (Lane 3) *Atseor 1-1* with *Atseor 1-1* flanking primers. (Lane 4) WT with *Atseor 1-1* flanking primers. (Lane 5) *Atseor 2-1* with *Atseor 2-1* specific T-DNA primer and flanking primer. (Lane 6) WT with *Atseor 2-1* specific T-DNA primer and Flanking primer. (Lane 7) *Atseor 2-1* with *Atseor 2-1* flanking primers. (Lane 8) WT with *Atseor 2-1* flanking primers.

**FIGURE 3:**
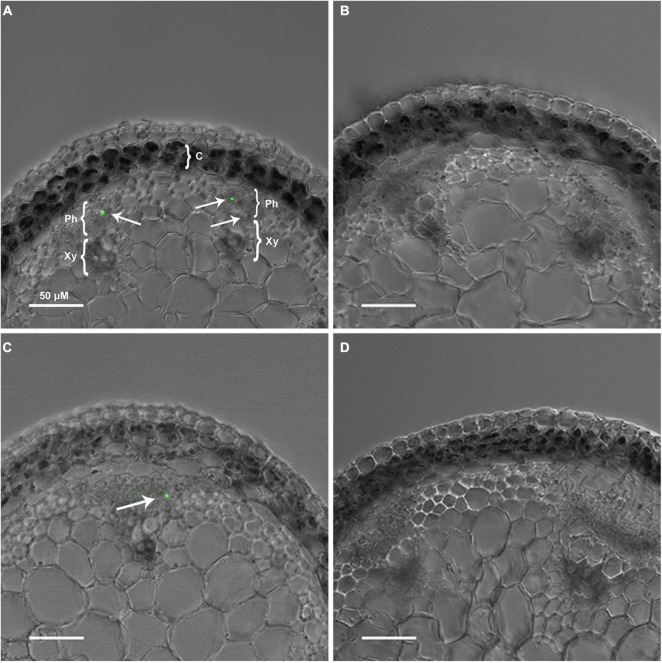
OGA ^488^ labeling of P-proteins (arrows) in *Arabidopsis thaliana* ecotypes. **(A)** Col-0-WT labeled with OGA ^488^. C, cortex; Ph, phloem; X, xylem, scale bar = 50 μm. **(B)** Col-0-WT with no probe (control). **(C)** GABI-KAT- 609F04 labeled with OGA ^488^. **(D)** SALK 148614C labeled with OGA ^488^. A minimum of three plants of each genotype was sectioned, and at least six sections probed and imaged. Number of labeled cells within individual vascular bundles: Col-0-WT mean 1.2, SE 0.1; GABI-KAT- 609F04 mean 1.1, SE 0.1; SALK 148614C mean 0, SE 0.

We next tested the hypothesis that the large intracellular bodies labeled within the secondary phloem were P-protein bodies within sieve elements. To investigate this possibility, OGA^488^ was tested for fluorescent staining in cross sections of Arabidopsis inflorescence stems from T-DNA insertion lines previously shown to disrupt the function of P-Protein encoding genes *AtSOER1* (AT3G01680) and *AtSEOR2* (AT3G01670), and wild type controls in the same ecotype background (Columbia). As shown in Figure 2, Arabidopsis lines homozygous for T-DNA insertions were first identified and confirmed using PCR (see section “Materials and Methods”). Fresh inflorescence stem cross sections were prepared and stained with OGA^488^ and visualized with laser confocal microscopy (see section “Materials and Methods”). As shown in Figure 3A, cross sections of wild type stems showed distinct, punctate staining of globular bodies within individual cells of the primary phloem in vascular bundles. The limited number of cells with staining was consistent with the modest amount of primary phloem and number of sieve elements in Arabidopsis inflorescence stems in comparison to the secondary phloem of poplar. No signal or background fluorescence was detected for wild type stems without OGA^488^ label (Figure 3B), indicating highly specific staining. Staining of T-DNA insertion line GABI-KAT 609F04 disrupting AtSEOR1 with OGA^488^ showed similar results as wildtype, with punctate staining in the primary phloem (Figure 3C). In contrast, no OGA^488^ staining was detected for T-DNA insertion line SALK 148614C disrupting AtSEOR2 (Figure 3D). We thus conclude that OGA^488^ staining is the result of interactions specific to AtSEOR2, and that OGA^488^ does not interact with AtSEOR1.

**FIGURE 4:**
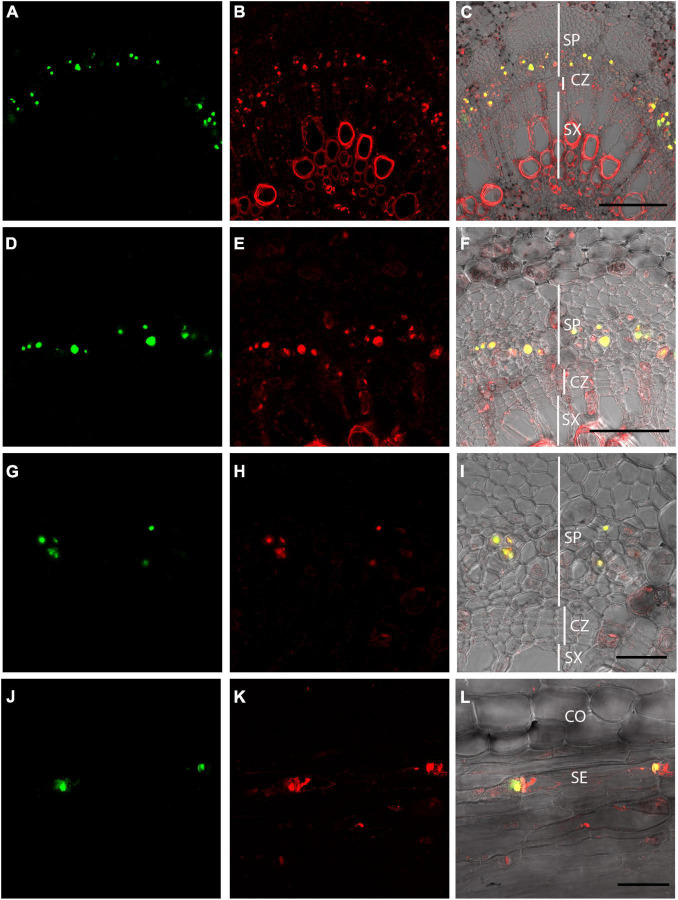
Dual labeling of P-proteins with OGA ^488^ and SR101 in *P. tremula* × *P. alba* (717) stem sections. **(A–C)** Poplar stem transverse section labeled with OGA ^488^ (green signal in **A**) and SR101 (red signal in **B**). Overlay **(C)** shows OGA^488^ signal colocalizes with SR101 within the secondary phloem (yellow) but SR101 stains additional cells and structures. Similar results are shown for increasing magnification in **(D–I)**. **(J–L)** Poplar longitudinal stem labeled with OGA^488^ (green signal in **J**) and SR101 (red signal in **K**) showing colocalization within sieve elements P-proteins near sieve plates. SR101 also stains additional structures in other cell types **(K,L)**. CO, cortex; SE, sieve element; CZ, cambial zone; SP, secondary phloem; SX, secondary xylem. Scale bars: **(A–C)** 100 μm, **(D–F)** 50 μm, **(G–L)** 20 μm.

OGA^488^ staining was compared to a previously described dye, SR101, shown to stain P-protein containing forisomes in *Vicia faba* (Peters et al., 2006). Double labeling of cross sections of poplar stems with OGA^488^ and SR101 is shown for increasing magnifications in Figure 4. OGA^488^ staining (Figures 4A–G) showed punctate labeling of individual cells within secondary phloem. SR101 labeling (Figures 4B–H) also showed punctate signals from individual cells in secondary phloem, but additionally labeled secondary cell walls within secondary xylem, as well as cytoplasm and cell wall constituents in cells of the cortex, rays, and primary xylem. Overlay of OGA^488^ and SR101 images (Figures 4C–I) showed overlapping signal for OGA^488^ and SR101. Thus, SR101 labeling provided additional support for the notion that OGA^488^ labels P-proteins, and also shows that OGA^488^ is highly specific in labeling P-protein bodies in contrast to this alternative probe. Longitudinal sections of poplar stems similarly showed very specific labeling of P-proteins with OGA^488^ overlapping with SR101, but with higher specificity. P-protein aggregates were labeled near sieve plates (Figure 4L), likely indicating injury of the labeled sieve elements during sectioning. Longitudinal sections also inform the interpretation of cross sections, where detection of P-protein aggregates is limited to the plane of optical sectioning.

**FIGURE 5:**
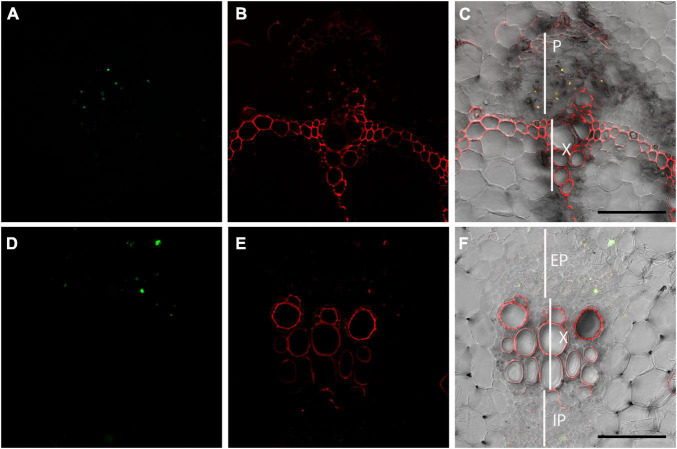
Dual labeling of P-proteins with OGA ^488^ and SR101 in snap bean and cucumber transverse stem sections. **(A–C)** Transverse sections of snap bean (*Phaseolus vulgaris*) labeled with OGA^488^ (green signal in **A**) and SR101 (red signal in **B**) shows colocalization within the phloem (yellow signal in **C**) but SR101 labels additional cell walls within xylem and phloem. **(D–F)** Transverse sections of cucumber (*Cucumis sativus*) labeled with OGA^488^ (green signal in **D**) and SR101 (red signal in **E**) shows colocalization within the external phloem (yellow signal in **C**) but SR101 labels additional cell walls within xylem and phloem. EP, external phloem; IP, internal phloem; P, phloem; X, xylem. Scale bars 100 μm.

To further test the ability of OGA^488^ to specifically stain P-proteins in additional species, double labeling of stems of *Phaseolus vulgaris* and *Cucumis sativus* was tested using OGA^488^ and SR101 (Figure 5). Similar to poplar and Arabidopsis, cross sections of both *P. vulgaris* (Figure 5A) and *C. sativus* (Figure 5D) had highly localized labeling of cells within the phloem but no other regions with OGA^488^, while SR101 labeled similar structures but also labeled secondary cell walls of xylem and other cytoplasmic components (Figures 5B,E). Overlay of OGA^488^ and SR101 signals showed good colocalization, again suggesting that OGA^488^ shows high specificity and can be used across a variety of species.

## Discussion

We found that the OGA^488^ reciprocal oligosaccharide probe gave reproducible and highly specific staining of P-protein bodies in poplar, Arabidopsis, snap bean and cucumber stems. This was an unexpected result, as the OGA^488^ probe was designed to reciprocally bind to chitin, and plant cells do not make chitin. Using previously characterized loss of function mutants for Arabidopsis P-protein components *AtSEOR1* and *AtSEOR2*, we found that AtSEOR2 is responsible for binding OGA^488^. As discussed below, our results provide a new method for rapidly labeling P-protein bodies in sectioned material and raise questions about the significance of carbohydrate binding in the function of P-protein bodies.

Labeling of P-protein bodies using OGA^488^ was robust and easy to execute in Arabidopsis, poplar, snap bean and cucumber stems. It should be noted that in the experiments here, freshly sectioned stem material was used and thus the morphology of P-proteins would be as expected for wounded phloem, and does not reflect the native morphology expected for intact, living cells. The labeling of P-protein bodies with OGA^488^ colocalized with a previously described labeling method using SR101 (Peters et al., 2006). The OGA^488^ labeling was highly specific for P-proteins in poplar (Figure 4) bean and cucumber (Figure 5), while SR101 also labeled additional structures non-specifically. Additionally, the probe proved to be stable in our hands, allowing the same staining solution to be reused multiple times without obvious degradation of signal or change in specificity. It should be noted that staining here was in sectioned stem tissues. The morphology of the P-protein bodies stained thus likely represent what is seen when the phloem is wounded and pressure is relieved, and thus does not reflect the native form of P-protein bodies in unwounded tissues. We did not succeed in labeling P-proteins robustly in intact (unsectioned) Arabidopsis roots using OGA^488^ (data not shown), suggesting the probe is not cell permeant or at least not capable of penetrating multiple cell layers to reach the phloem. This limitation could potentially be overcome by those wanting to achieve high resolution imaging of native P-protein morphology using OGA^488^ by partial dissection of tissues (e.g., Peters et al., 2006), and/or new approaches for delivery of cell-impermeable probes into live cells (e.g., Zhang et al., 2019). However, the ease of the assay described here could be extended to many applications and questions not requiring native P-protein morphology including identification of sieve elements, or how P-protein bodies change in different genotypic backgrounds, in response to insect or pathogen interactions, or assays of physical wounding or biochemical responses. This is in contrast to other technically challenging approaches (Truernit, 2019) designed to maintain P-protein integrity for immunolocalization and/or electron microscopy (Hunziker and Schulz, 2019), or transgenic expression of GFP-translational fusions for live cell imaging (Pélissier et al., 2008; Cayla et al., 2019).

The target of labeling OGA^488^ was investigated here using previously described knockout mutants for Arabidopsis P-protein-encoding *AtSEOR1* and *AtSEOR2* (Anstead et al., 2012). Signal for OGA^488^ was present in *atseor1* mutants but lacking in *atseor2* mutants, allowing us to conclude that AtSEOR2 binds OGA^488^. How OGA^488^ labels AtSEOR2 is an intriguing question, and while not directly addressed in the present work it is possible that binding is through the reciprocal carbohydrate bonding approach originally designed for the probe (Mravec et al., 2014), which would predict that AtSEOR2 is a glycoprotein decorated with carbohydrate capable of interacting with OGA^488^. Previous reports investigating the structure and function of P-proteins ascribed *in vitro* carbohydrate-binding lectin activity to Phloem Protein 2 (PP2) from *Cucurbita maxima* and *Cucumis melo*, which bound to chitin columns (Sabnis and Hart, 1978; Allen, 1979; Read and Northcote, 1983; Dinant et al., 2003). However, PP2-like proteins are phylogenetically unrelated to the AtSEOR2, and AtSEOR2 has not been reported to be glycosylated or routed through the secretory pathway. Alternatively, the AtSEOR2 protein may selectively bind the probe through protein-carbohydrate interactions. To our knowledge, carbohydrate-binding lectin properties have not been previously described for AtSEOR2 and could now be further investigated in future research.

It is intriguing to ask what the functional significance of lectin-like carbohydrate binding of AtSEOR2 to OGA^488^ could be. Previous research has suggested various functions of P-proteins, including directly interacting with or otherwise aggravating insect, fungal, or viral pests of the phloem, but irrefutable assignment of function to P-proteins remains elusive (Knoblauch et al., 2014). Lectin-like carbohydrate binding by AtSEOR2 to the OGA^488^ could be fortuitous, or may reflect a normal function of the protein. Chitin present in the stylets of phloem-feeding insects and the wall of fungal pathogens both present potential binding targets for AtSEOR2.

The biological complexity and extreme economic costs of phloem-feeding insects and associated vectored pathogens (Jiang et al., 2019) requires new tools, concepts and insights. We hope that the labeling procedure reported here will be a useful addition to the tools available for the study of P-proteins, and inspire new research into the nature of carbohydrate binding by AtSERO2.

## Data Availability Statement

The original contributions presented in the study are included in the article/supplementary material, further inquiries can be directed to the corresponding author/s.

## Author Contributions

AG conceived and managed the study. AL made the initial observation of OGA488 labeling. LS and AL genotyped Arabidopsis lines. PA performed imaging. AG and PA drafted the manuscript. All authors contributed to the article and approved the submitted version.

## Conflict of Interest

The authors declare that the research was conducted in the absence of any commercial or financial relationships that could be construed as a potential conflict of interest.

## Publisher’s Note

All claims expressed in this article are solely those of the authors and do not necessarily represent those of their affiliated organizations, or those of the publisher, the editors and the reviewers. Any product that may be evaluated in this article, or claim that may be made by its manufacturer, is not guaranteed or endorsed by the publisher.
